# Our New York Letter

**Published:** 1888-11

**Authors:** 


### Correspondence.

### OUR NEW YORK LETTER.

DISTINGUISHED MEDICAL VISITORS—RECEPTION TO PROF. ESMARCH—DR. SAYRE'S CLINIC AT BELLEVUE HOSPITAL—ESMARCH ON ARTICULAR NEUROSES—THE NEW PROFESSOR OF ANATOMY AT THE COLLEGE OF PHYSICIANS AND SURGEONS.

*To the Editors Atlanta Medical and Surgical Journal:*

The long vacation is now practically over, and during the present month there will be a steady flow of those returning physicians and laymen who are among the latest to leave for home. The session at the College of Physicians and Surgeons commenced on the first of the month and the lectures have begun at the other medical schools also.

At present we have quite a number of distinguished visitors in town, among which may be mentioned Sir William McCormack and Dr. Wm. Ord, of St. Thomas Hospital, London; Mr. Arthur Durham, of Guy's Hospital, London; Mr. Reginald Harrison, Dr. Graily Hewitt and Professor Esmarch. Last month a reception was given to Dr. Graily Hewitt by Dr. W. Gill Wylie at his private hospital.

One of the pleasantest social affairs of the season occurred a few nights ago, when a reception was tendered Professor Esmarch by Dr. Robert F. Wier and Dr. Wm. T. Bull. The gathering took place in the parlors of the New York Hospital, which spacious and handsome apartments were especially suitable for an occasion of this kind. Dr. Esmarch is regarded by many as the first military surgeon of the world, and the reception brought together nearly all the prominent physicians and surgeons of this and neighboring cities. The professor is rather a conspicuous looking person of medium height, with large, gray eyes and a long, white beard. The evening was spent in a very informal manner and scientific discussions were for the most part omitted. All present were presented to the venerable guest, and refreshments were liberally supplied, but there were no speeches nor toasts, the time being all spent in social conversation. While staying in New York Professor Esmarch is the guest of Dr. F. Lange. There is a great desire here to see him operate, but it is hardly probable that he will accept all the invitations in this direction from the various hospitals. He has already operated at the New York Hospital on a patient with lymphatic tumors of the neck. Dr. L. A. Stimson, in introducing him, said that he was the one man who had made the greatest advances in recent years in the treatment of gunshot wounds. It is needless to say that the operation, although not a very formidable one, was performed with much skillful ease and thoroughness, but however simple an operation may be, the desire to see just how Esmarch will perform it is very great.

Dr. Lewis A. Sayre's clinic was honored the other day by the presence of Mr. Reginald Harrison and Mr. Durham. This clinic is held at Bellevue Hospital, and it was announced that Mr. Harrison would do a perineal section. It was stated later, however, that the patient had had a chill, so the operation was omitted, in place of which Mr. Harrison entertained those present by a description of his operation for prostatotomy. Mr. Durham gave the details of a case in which he performed pharyngotomy at Guy's Hospital. The patient in this instance had swallowed the handle of a jug. A patient was then brought in suffering from beginning hip disease, and Dr. Sayre, after describing the case, applied the extension splint, known by his name, and then closed the clinic with a few appropriate remarks.

At the last meeting of the Medico-Chirurgical Society of German Physicians, Professor Von Esmarch was present and spoke on the subject of articular neuroses. It was expected that he would read a paper, but not having prepared one, remarks on the above topic were given. Articular neuroses, he said, had been described also under the names of hysterical joint affections and neuralgia of joints. As neuralgic pain was often absent, he preferred the name neurosis. In a typical case which he saw, the patient, a woman, appeared with a swelling of the leg, and as it had existed so in a very painful state for years, and was getting no better, she desired an amputation. In appearance it resembled an ordinary white tumor. A cure was effected in this case by inducing the woman to rise and walk. Among the symptoms of joint neurosis were contractures, scoliosis, dropsy, temporary puffiness, and also wheals resembling urticaria. Symptoms which occur in numerous other diseases might be present. A traumatism was often the exciting cause, and if the true nature of the trouble were not discovered, it usually kept on getting worse. He also mentioned uterine disease as a cause and spoke of a case which closely resembled morbus coxarius. The diagnosis, he said, was often difficult, but in this disease there was generally a great disproportion between subjective and objective symptoms. Localized tenderness was generally well marked, and where the patient steadily grew worse under rational treatment for some supposed joint trouble, it was very suggestive of articular neurosis. This disease had also, he said, been recognized by a peculiar expression of the face or a neurotic countenance. Moral suasion formed the most important treatment. Exercise was useful and also cold bathing. In the discussion which followed, Dr. Wm. Detmold spoke of a case of neurosis of the elbow-joint which he considered typical. The patient would cry with pain when the physician's hand was several inches away from the joint. Dr. Lange remarked that true pathological lesions, such as atrophy from disease, might occur. He had observed articular neurosis in young men, especially masturbators, and also business men subjected to much worriment. In closing Dr. Esmarch said that soms patients only felt a weakness in the limbs. A characteristic of these joint affections was that, by diverting the patient's attention, pressure might be made on the tender spots above referred to without causing pain.

The chair of anatomy at the College of Physicians and Surgeons, left vacant by the death of Prof. Sabine, has been filled by

Dr. R. J. Hall. At the same time Dr. Charles McBurney received an appointment as Adjunct Lecturer on Surgery.

Dr. Wm. T. Bull, who has, during the summer, remained in town attending to an extensive hospital and private practice, sailed for Europe a few days ago, expecting to return about December 1. A. F.

*New York, Oct. 8, 1888.*

